# Supplementary material for: Nature-Based Rehabilitation for Patients with Long-Standing Stress-Related Mental Disorders: A Qualitative Evidence Synthesis of Patients’ Experiences
Source: Int J Environ Res Public Health. 2021 Jun 27;18(13):6897. doi: 10.3390/ijerph18136897 (PMC8297286; doi:10.3390/ijerph18136897)
Supplement: Supplementary file 1 [file ijerph-18-06897-s001.zip › Table S1 Search strategies and results.pdf]

Table S1 Search strategies and results

Database: PubMed
Date: 17 Dec 2020
No. of results: 178 records

Search	Query	Results
**#39**	**Search: #22 AND #38 Sort by: Most Recent**	**178**
#38	Search: ((semi-structured[tiab] OR semistructured[tiab] OR unstructured[tiab] OR informal[tiab] OR in-depth[tiab] OR indepth[tiab] OR face-to-face[tiab] OR structured[tiab] OR guide[tiab] OR guides[tiab]) AND (interview*[tiab] OR discussion*[tiab] OR questionnaire*[tiab])) OR focus group[tiab] OR focus groups[tiab] OR qualitative[tiab] OR ethnograph*[tiab] OR fieldwork[tiab] OR field work[tiab] OR key informant[tiab] OR experience*[tiab] OR narrative[tiab] OR "Interviews as Topic"[Mesh] OR "Focus Groups"[Mesh] OR "Narration"[Mesh] OR "Qualitative Research"[Mesh] OR "Personal Narratives as Topic"[Mesh] Sort by: Most Recent	1,463,968
#22	Search: #14 NOT #15 Filters: Danish, English, Norwegian, Swedish Sort by: Most Recent	810
#21	Search: #14 NOT #15 Filters: Danish, English, Norwegian Sort by: Most Recent	810
#20	Search: #14 NOT #15 Filters: English, Norwegian Sort by: Most Recent	809
#19	Search: #14 NOT #15 Filters: English Sort by: Most Recent	808
#16	Search: #14 NOT #15 Sort by: Most Recent	853
#15	Search: ((eukaryota[mh]) NOT (eukaryota[mh] AND humans[mh])) Sort by: Most Recent	5,457,993
#14	Search: #10 OR #13 Sort by: Most Recent	1,232
#13	Search: #11 AND #12 Sort by: Most Recent	220
#12	Search: treatment OR rehabilitation OR therapy OR therapies Sort by: Most Recent	11,662,594
#11	Search: nature-based[tiab] OR nature-supported[tiab] OR nature-assisted[tiab] Sort by: Most Recent	674
#10	Search: #6 AND #9 Sort by: Most Recent	1,072
#9	Search: #7 OR #8 Sort by: Most Recent	13,007
#8	Search: nature-based[tiab] OR nature-supported[tiab] OR nature-assisted[tiab] OR gardening[tiab] OR garden[tiab] OR horticultural[tiab] OR horticulture[tiab] Sort by: Most Recent	13,003
#7	Search: "Horticultural Therapy"[Mesh] Sort by: Most Recent	67
#6	Search: #2 OR #3 OR #4 OR #5 Sort by: Most Recent	1,924,722
#5	Search: pain[tiab] OR anxiety[tiab] OR depression[tiab] OR fatigue[tiab] OR insomnia[tiab] OR sleep disturbance*[tiab] Sort by: Most Recent	1,162,418
#4	Search: stress[tiab] OR exhaustion[tiab] OR stress-related[tiab] OR stress-induced[tiab] OR adjustment disorder*[tiab] OR adaptation syndrome[tiab] OR burnout[tiab] OR burn-out[tiab] Sort by: Most Recent	808,964
#3	Search: "Adjustment Disorders"[Mesh] Sort by: Most Recent	4,232
#2	Search: "Stress, Psychological"[Mesh] Sort by: Most Recent	134,372

Database: Embase 1974 to 2020 December 15 (OvidSP)
Date: 17 Dec 2020
No. of results: 148 records

#	Searches	Results
1	exp job stress/	10504
2	exp mental stress/	84589
3	exp burnout/	20077
4	exp adjustment disorder/	3904
5	(stress or exhaustion or stress-related or stress-induced or adjustment disorder$ or adaptation syndrome or burnout or burn-out or pain or anxiety or depression or fatigue or insomnia or sleep disturbance$).ab,kw,ti.	2588864
6	1 or 2 or 3 or 4 or 5	2625884
7	exp horticultural therapy/	119
8	(nature-based or nature-supported or nature-assisted or gardening or garden or horticultural or horticulture).ab,kw,ti.	14285
9	7 or 8	14298
10	6 and 9	1282
11	(nature-based or nature-supported or nature-assisted).ab,kw,ti.	785
12	(treatment or rehabilitation or therapy or therapies).af.	11747016
13	11 and 12	234
14	10 or 13	1450
15	exp eukaryote/	27396745
16	human.sh.	21610630
17	15 and 16	21610630
18	15 not 17	5786115
19	14 not 18	972
20	limit 19 to (embase or medline)	733
21	limit 20 to (danish or english or norwegian or swedish)	663
22	(("semi-structured" or semistructured or unstructured or informal or "in-depth" or indepth or "face-to-face" or structured or guide) adj3 (interview* or discussion* or questionnaire*)).ti,ab. or (focus group* or qualitative or ethnograph* or fieldwork or field work or key informant or experience* or narrative).tw,kw. or qualitative research/	1921086
**23**	**21 and 22**	**148**

Database: CINAHL; AMED - The Allied and Complementary Medicine Database; APA PsycInfo via EbscoHost
Date: 17 Dec 2020
No. of results: 287 records

#	Query	Result
**S9**	**S6 AND S7**<br>**Language: - english**	**287**
S8	S6 AND S7	296
S7	AB (((semi-structured OR semistructured OR unstructured OR informal OR in-depth OR indepth OR face-to-face OR structured OR guide OR guides) AND (interview* OR discussion* OR questionnaire*)) OR ”focus group” OR ”focus groups” OR qualitative OR ethnograph* OR fieldwork OR ”field work” OR ”key informant” OR experience* OR narrative) OR TI (((semi-structured OR semistructured OR unstructured OR informal OR in-depth OR indepth OR face-to-face OR structured OR guide OR guides) AND (interview* OR discussion* OR questionnaire*)) OR ”focus group” OR ”focus groups” OR qualitative OR ethnograph* OR fieldwork OR ”field work” OR ”key informant” OR experience* OR narrative) OR SU (((semi-structured OR semistructured OR unstructured OR informal OR in-depth OR indepth OR face-to-face OR structured OR guide OR guides) AND (interview* OR discussion* OR questionnaire*)) OR ”focus group” OR ”focus groups” OR qualitative OR ethnograph* OR fieldwork OR ”field work” OR ”key informant” OR experience* OR narrative)	1,615,747
S6	S4 NOT S5	804
S5	((MH "Plants+") NOT (MH "Plants+" AND MH "Human"))	84,063
S4	S1 OR S2 OR S3	843
S3	TI ((stress OR exhaustion OR stress-related OR stress-induced OR adjustment disorder* OR adaptation syndrome OR burnout OR burn-out OR pain OR anxiety OR depression OR fatigue OR insomnia OR sleep disturbance*) AND (nature-based OR nature-supported OR nature-assisted OR Gardening OR garden OR horticultural OR horticulture)) OR ((nature-based OR nature-supported OR nature-assisted) AND (treatment OR rehabilitation OR therapy OR therapies))	250
S2	AB ((stress OR exhaustion OR stress-related OR stress-induced OR adjustment disorder* OR adaptation syndrome OR burnout OR burn-out OR pain OR anxiety OR depression OR fatigue OR insomnia OR sleep disturbance*) AND (nature-based OR nature-supported OR nature-assisted OR Gardening OR garden OR horticultural OR horticulture)) OR ((nature-based OR nature-supported OR nature-assisted) AND (treatment OR rehabilitation OR therapy OR therapies))	729
S1	((MW Stress OR MW exhaustion OR MW Stress-related OR MW stress-induced OR MW adjustment disorder* OR MW adaptation syndrome OR MW burnout OR MW burn-out OR MW pain OR MW anxiety OR MW depression OR MW fatigue OR MW insomnia OR MW sleep disturbance*) AND (MW nature-based OR MW nature-supported OR MW nature-assisted OR MW Gardening OR MW garden OR MW horticultural OR MW horticulture)) OR ((MW nature-based OR MW nature-supported OR MW nature-assisted) AND (MW treatment OR MW rehabilitation OR MW therapy OR MW therapies))	145

Database: The Cochrane Library
Date: 17 Dec 2020
No. of results: 23 records
Cochrane reviews: 2
Trials: 21

ID	Search	Hits
#1	(stress or exhaustion or stress-related or stress-induced or adjustment disorder* or adaptation syndrome or burnout or burn-out or pain or anxiety or depression or fatigue or insomnia or sleep disturbance*):ti,ab,kw (Word variations have been searched)	352336
#2	(nature-based or nature-supported or nature-assisted or gardening or garden or horticultural or horticulture):ti,ab,kw (Word variations have been searched)	557
#3	#1 AND #2	174
#4	(nature-based or nature-supported or nature-assisted):ti,ab,kw (Word variations have been searched)	84
#5	(treatment or rehabilitation or therapy or therapies):ti,ab,kw (Word variations have been searched)	1012047
#6	#4 AND #5	58
#7	#3 OR #6	200
#8	(((semi-structured OR semistructured OR unstructured OR informal OR in-depth OR indepth OR face-to-face OR structured OR guide OR guides) AND (interview* OR discussion* OR questionnaire*)) OR "focus group" OR "focus groups" OR qualitative OR ethnograph* OR fieldwork OR "field work" OR "key informant" OR experience* OR narrative):ti,ab,kw (Word variations have been searched)	158877
#9	#7 AND #8	52
#10	(clinicaltrials or trialsearch):so	351637
**#11**	**#9 NOT #10**	**23**
