# Supplementary material for: Nature-Based Rehabilitation for Patients with Long-Standing Stress-Related Mental Disorders: A Qualitative Evidence Synthesis of Patients’ Experiences
Source: Int J Environ Res Public Health. 2021 Jun 27;18(13):6897. doi: 10.3390/ijerph18136897 (PMC8297286; doi:10.3390/ijerph18136897)
Supplement: Supplementary file 1 [file ijerph-18-06897-s001.zip › Table S2 List of excluded studies.pdf]

Table S2 List of excluded studies, with reason for exclusion

Authors	Year	Title	Reason for exclusion
Adams M, Morgan J	2018	Mental health recovery and nature: How social and personal dynamics are important	Unclear population: mainly patients with depression or anxiety
Barley E, Robinson S, Sikorski J	2012	Primary-care based participatory rehabilitation: Users' views of a horticultural and arts project	Wrong population: mainly depression
Bradley CJ	1998	Applied ecopsychology: The experience of treatment when the treatment method is nature-based psychotherapy	Wrong population: various psychological problems, eg marital problems – not stress-related disorders; wrong exposure: nature-based psychotherapy
Carlson K, Kingsley J, Strimaitis C, Birnbaum S, Quinn T, Espinoza R, Rapaport A	2020	Nature-based Group Therapy Reflections: A Grounded Theory Study	Wrong population: major depression, post-traumatic stress disorder, anxiety disorders, and co-occurring substance use disorders.
Cerwén G, Pedersen E, Pálsdóttir AM	2016	The Role of Soundscape in Nature-Based Rehabilitation: A Patient Perspective	Wrong focus: Looks at how participants perceive different types of sound.
Eriksson T, Karlström E, Jonsson H, Tham K	2010	An exploratory study of the rehabilitation process of people with stress-related disorders	Unclear population: stress-related disorder not separable from other conditions; wrong focus: Rehabilitation with a cognitive approach, of which an undefined proportion of participants had undergone rehabilitation with a nature component.
Eriksson T, Westerberg Y, Jonsson H	2011	Experiences of women with stress-related ill health in a therapeutic gardening program	Wrong population: mainly depression, anxiety and acute stress reaction
Harris H	2017	The social dimensions of therapeutic horticulture	Wrong population: mental distress, stress-related disorder not specified; wrong exposure: not group-based rehabilitation
Howarth M, Rogers M, Withnell N, McQuarrie C	2018	Growing spaces: An evaluation of the mental health recovery programme using mixed methods	Unclear population: people with mental health problems, stress-related disorders not specified
Maund PR, Irvine KN, Reeves J, Strong, E, Cromie R, Dallimer M, Davies ZG	2019	Wetlands for Wellbeing: Piloting a Nature-Based Health Intervention for the Management of Anxiety and Depression	Wrong population: depression and/or anxiety
O'Brien L	2018	Engaging with and Shaping Nature: A Nature-Based Intervention for Those with Mental Health and Behavioural Problems at the Westonbirt Arboretum	Wrong population: not stress-related disorder; wrong exposure: general gardening, not an NBR programme
Sahlin E, Ahlborg Jr G, Matuszczyk JV, Grahn P	2014	Nature- based stress management course for individuals at risk of adverse health effects from work-related stress-effects on stress related symptoms, workability and sick leave	Wrong population: not long-term stress-related disorder
Siu AMH, Kam M, Mok I	2020	Horticultural Therapy Program for People with Mental Illness: A Mixed-Method Evaluation	Wrong population: mental illness, not stress-related disorder
Wästberg BA, Harris U, Gunnarsson AB	2020	Experiences of meaning in garden therapy in outpatient psychiatric care in Sweden. A narrative study	Unclear population: common mental disorders, stress-related disorders not specified
